# Molecular Events Occurring During Softening of Strawberry Fruit

**DOI:** 10.3389/fpls.2019.00615

**Published:** 2019-05-15

**Authors:** Maria Alejandra Moya-León, Elena Mattus-Araya, Raul Herrera

**Affiliations:** Instituto de Ciencias Biológicas, Universidad de Talca, Talca, Chile

**Keywords:** strawberry, cell wall, softening, transcription factor, fruit ripening

## Abstract

Changes in fruit texture taking place during ripening, described as softening, are mainly due to alterations in structure and/or composition of the cell wall. Several non-covalent interactions between the three carbohydrate polymers of the cell wall, cellulose, pectins and hemicellulose, and many structural proteins and ions, enable a complex structure. During softening, the disassembly of the cell wall structure takes place, mediated by a complete set of cell wall degrading enzymes or proteins. Softening is a coordinated event that requires the orchestrated participation of a wide variety of proteins. Plant hormones and a set of transcription factors are the organizers of this multi-protein effort. Strawberry is a non climacteric fruit that softens intensively during the last stages of development. The Chilean strawberry fruit (*Fragaria chiloensis*), the maternal relative of the commercial strawberry (*F.* × *ananassa*), softens even faster than commercial strawberry. Softening of the Chilean strawberry fruit has been studied at different levels: changes in cell wall polymers, activity of cell wall degrading enzymes and transcriptional changes of their genes, providing a general view of the complex process. The search for the ‘orchestra director’ that could coordinate softening events in strawberry fruit has been focussed on hormones like ABA and auxins, and more precisely the relation ABA/AUX. These hormones regulate the expression of many cell wall degrading enzyme genes, and this massive transcriptional change that takes place involves the participation of key transcriptional factors (TF). This review provides an update of the present knowledge regarding the softening of strawberry fruit. Nevertheless, the entire softening process is still under active research especially for the great influence of texture on fruit quality and its high impact on fruit shelf life, and therefore it is expected that new and promising information will illuminate the field in the near future.

## Fruit Ripening

Fruit ripening is a complex, genetically programmed and environmentally regulated process (Giovannoni, 2001, 2004), and explained by a series of biochemical and physiological changes that take place at the terminal stage of fruit development. It has a remarkable impact on fruit quality, post-harvest life and consumer acceptance. Several modifications that take place during ripening cause changes in color, texture, flavor, aroma and nutritional value, and all these alterations will transform fleshy fruit attractive and palatable for consumers. Biochemical processes associated with ripening include degradation of chlorophyll and starch, biosynthesis of pigments and volatile compounds, accumulation of sugars and organic acids (Giovannoni, 2001). Ripening associated traits are governed by external (i.e., light, temperature) and internal factors (i.e., gene regulation, hormonal control), which are integrated within the fruit and allow the development of ripe fruit attributes. Although ripening changes are expected in a commodity, some of them have a negative impact on its quality; for example, fruit with a rapid softening will display a shorter shelf-life and higher pathogen susceptibility than a harder one, and therefore intensive and sometimes injurious horticultural management conditions will be required to extend its post-harvest life. Delaying negative traits remained a major challenge, and for this reason, it is essential to understand key control points of the global ripening process.

## Fruit Softening

Softening is one of the most important ripening traits. Softening rate not only determines post-harvest shelf life but also other economically important aspects, such as the frequency of harvesting, handling procedures and the distance that the fruit can be transported. From the consumer’s point of view, texture is the main quality attribute that determines fruit acceptance in the market (Goulao and Oliveira, 2008).

In general, the main causes of fruit softening are cell wall disassembly and the reduction of cell to cell adhesion, as a result of middle lamella dissolution (Brummell and Harpster, 2001; Brummell, 2006). Cell wall modifications include depolymerization of the glycan matrix, solubilization and/or depolymerization of pectins, and loss of neutral sugars from pectin side chains (Figueroa et al., 2008; Goulao and Oliveira, 2008). These changes also induce the loosening of the xyloglucan-cellulose network (arabinan and galactan side chains from rhamnogalacturonan I) and cell wall swelling, increasing wall porosity that may facilitate the access of degradative enzymes to their substrates (Brummell, 2006; Paniagua et al., 2017). Finally, cell wall modifications take place in most fleshy fruit, independent of its classification as climacteric or non-climacteric, however, softening rate and intensity varies from fruit to fruit.

## Cell Wall Modifying Enzymes

Polysaccharide modifications can alter cell wall properties and are largely due to the sequential and coordinated action of a variety of ripening-related enzymes, which are secreted into the cell wall space during the progress of ripening (Opazo et al., 2010). Some of these enzymes are present throughout fruit development, others increase or decrease during development, whereas those related to softening appear only during ripening (Brummell and Harpster, 2001; Brummell, 2006). The entire set of enzymes can modify the structure of cell wall polysaccharides by removing sequentially the side chains of ramified polysaccharides and breaking down the main bone of each polysaccharide. As a result of this, covalent bonds are broken down promoting a reduction in the size of polysaccharides and the degree of polymerization, but also the non-covalent interactions between the polysaccharides are also modified. Specific changes include cleavage of polymeric backbones, removal of polymeric or single-sugar side chains, elimination of methyl ester or acetyl groups from homogalacturonan (HGA), and loosening of hydrogen bonds between cellulose microfibrils and glycans (Paniagua et al., 2014, 2017). The addition of all these minor effects has led to extensive softening and ultimately tissue disintegration.

The disassembly of the complex cell wall structure requires the orderly participation of enzymes. Each cell wall degrading enzyme displays a particular activity (hydrolase, transglycosylase, lyase) and needs to recognize its own substrate within the cell wall structure. After the action of the first set of enzymes, hidden substrates can be exposed to second round enzymes. In addition, some proteins without activity also take part in the process. Although efforts have been made to gain in the understanding of the role of enzymes involved in fruit softening, still there are lots of enzymes present in fruit whose activity or transcript abundance has been described but its function in softening has not yet been reported.

The key to understand fruit softening lies in understanding the cell wall structure, its composition, as well as, its interaction with the different cell wall-degrading enzymes. During the past few years, significant advances in the knowledge of genes involved in fruit softening have provided good insight into the mechanism of cell wall disassembly. But, there is still a dearth and need in understanding the precise role of each polysaccharide and each enzyme involved in fruit softening. Another level of complexity of the process is the existence of isoforms for some of the enzymes involved in cell wall disassembly. The characterization of those enzymes and the time when they are expressed during fruit development is just coming up to light.

## Strawberries and Their Chilean Mother

*Fragaria chiloensis* (L.) Mill or the Chilean strawberry is a native fruit from Chile, distributed throughout the Andes and the coastal mountain range of Chile (Hancock et al., 1999; Lavín et al., 2000; Retamales et al., 2005). The berry is appreciated for its good organoleptic qualities, being its sweet and pleasant aroma the main characteristic of this non-climacteric fruit, in addition to its fruit size, resistance to pathogens and better sustainability to soil salinity and low temperature (González G. et al., 2009; González M. et al., 2009). Interestingly, *F. chiloensis* is the maternal relative of *Fragaria* × *ananassa*, the commercial strawberry, and therefore a good gene source in strawberry breeding programs. *F. chiloensis* has the potential to be developed as a new exotic berry in the world market (Retamales et al., 2005), however, the fruit is highly perishable as its rapid softening alters the texture and negatively influences its post-harvest life and quality (Perkins-Veazie, 1995; Figueroa et al., 2008). Hence, in order to reach the international market there is need for improvement of its shelf life. Nevertheless, the short life cycle and its ploidy (2n = 8X = 56) makes this *Fragaria* species a great challenge for any breeding initiative. In addition, *F. chiloensis* has not been sequenced yet as the challenge for sequencing octoploid species is much higher than for diploids ones. The only strawberry species sequenced so far is *Fragaria vesca* (Shulaev et al., 2010), also recognized as woodland strawberry, although we are aware that efforts are ongoing with the sequencing of *F.* × *ananassa*.

In strawberries, softening is characterized by an extensive dissolution of the middle lamella of the cortical parenchyma cells; cells appear disconnected by a considerable intercellular space and a little cell to cell contact area (Redgwell et al., 1997; Santiago-Doménech et al., 2008). At the cell wall level, a moderate pectin solubilization and depolymerisation, and a slight reduction of the molecular weight of hemicellulosic polymers are general features of softening (Koh and Melton, 2002; Nishizawa et al., 2002; Rosli et al., 2004; Figueroa et al., 2010). On the other hand, cellulose content appears to be small in strawberry fruit and remained almost unaffected during softening (Koh et al., 1997; Figueroa et al., 2008). Comparatively *F. chiloensis’s* fruit exhibited a higher softening rate than *F.* × *ananassa* (cv. Chandler) during ripening (Figueroa et al., 2010), and the most significant change observed in cell wall components is in pectin-rich fractions, especially in the HCl-soluble pectin fraction (i.e., covalently bound pectins), rather than in the hemicellulose fraction (Figueroa et al., 2010; Paniagua et al., 2017). This indicates that softening of strawberry fruit is closely related to catabolism of covalently bound pectins rather than ionically bound pectins or hemicelluloses.

Following this argument, softening studies in strawberry fruit have been focused mainly on pectinolytic enzymes, as pectin degradation has been proposed to be a major determinant of fruit firmness (Jiménez-Bermúdez et al., 2002; Rosli et al., 2004; Figueroa et al., 2008; Santiago-Doménech et al., 2008; Villarreal et al., 2008; Quesada et al., 2009). Nevertheless, just few years ago enzymes that may affect the hemicellulose/cellulose network have been incorporated in fruit studies (Nardi et al., 2014). How these enzymes take concerted part in cell wall disassembly? is still intriguing. At the present time, several studies have reported a series of enzymes involved in strawberry fruit softening, such as, Polygalacturonase [PG] (EC.3.2.1.15) (García-Gago et al., 2009; Quesada et al., 2009), Pectate lyase [PL] (EC.4.2.2.2) (Benítez-Burraco et al., 2003; Youssef et al., 2009), Pectin methylesterase [PME] (EC.3.1.1.11) (Castillejo et al., 2004; Osorio et al., 2008), β-Galactosidase [β-Gal] (EC.3.2.1.23) (Trainotti et al., 1999), α-Arabinofuranosidase [AFase] (EC.3.2.1.-) (Rosli et al., 2009), Endoglucanase [EGase] (EC.3.2.1.4) (Palomer et al., 2006; Mercado et al., 2010), and Xyloglucan endotransglycosylase/hydrolase [XTH] (EC 2.4.1.-) (Opazo et al., 2010, 2013), among others. Also proteins with no catalytic activity seem to be important, as the case of Expansins [EXP] (Harrison et al., 2001).

In the following paragraphs, we compiled evidence of key cell wall degrading enzymes in fruit softening, describing how the process could proceed and searching for key regulators of this biological process.

## Metabolism of Pectins

In strawberries, a reduction in the content of pectins extracted from cell wall material is advised during softening nevertheless significant differences have been reported between different species. In *F.* × *ananassa* the metabolism of pectins includes a reduction in the size of pectin-polymers especially in HCl-soluble pectin fraction (shift to lower MW polymers), while in *F. chiloensis* there are no major changes in the size of pectin-polymers that remained in the cell wall after softening (Figueroa et al., 2010), suggesting that pectins are fully depolymerized to soluble saccharides in *F. chiloensis* and partly fractionated in *F.* × *ananassa*. This important difference should be explained by a different set of pectinases.

**Figure 1:**
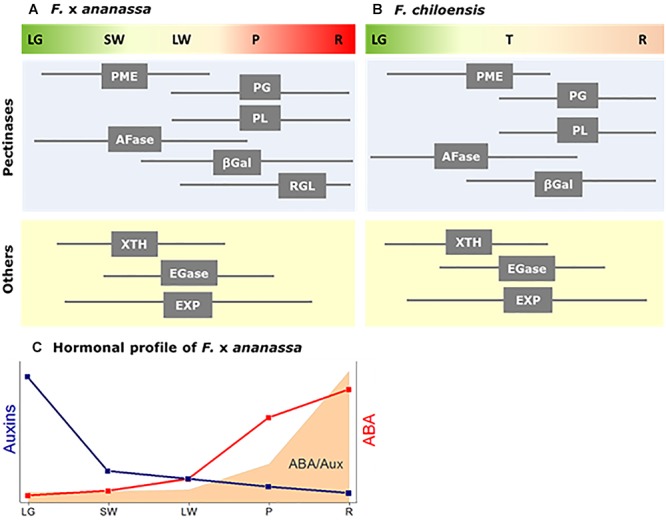
Diagram showing the temporal expression profile of cell wall degrading genes during ripening development of two strawberry fruit species: **(A)** *F.***×** *ananassa*, and **(B)** *F. chiloensis*. Cell wall degrading enzymes correspond to Pectin methylesterase (PME), α-Arabinofuranosidase (AFase), Polygalacturonase (PG), Pectate lyase (PL), β-Galactosidase (βGal), Xyloglucan endotransglycosilase/ hydrolase (XTH), Endoglucanase (EGase), Expansins (EXP) and Rhamnogalacturonan I lyase (RGL). Fruit stages for *F.***×***ananassa* correspond to: LG, large green; SW, small white; LW, large white; P, pink/turning; R, red. Fruit stages for *F. chiloensis* correspond to: LG, large green (large size fruit with red achenes); T, turning (large size fruit with white receptacle and red achenes); and R, ripe fruit (full size fruit with pink receptacle and red/brown achenes). **(C)** Scheme showing changes in auxins and ABA levels in *F.***×***ananassa* fruit; in gray the ABA/AUX ratio (adapted from Symons et al., 2012).

A group of enzymes linked to the metabolism of pectins are expressed in a coordinate way during the last stages of fruit development, corresponding to the ripening phase of strawberry (LG to R stages) (Figure 1). Pectinases, and particularly PG, have received extensive attention due to their direct relation to strawberry fruit firmness. PG catalyses the hydrolysis of alpha-1,4 glycosyl bonds between the galacturonic acid residues of homogalacturonan. In *F. chiloensis*, both *PG* transcripts and activity increase during ripening development of the fruit, reaching the highest activity at the ripe stage (Figueroa et al., 2008). In comparison, in different *F.* × *ananassa* cultivars *PG* transcripts accumulates during fruit development although changes in activity were not evident (Villarreal et al., 2008). Another study reported different levels of PG activity and *PG* transcripts in *F.* × *ananassa* cultivars with texture dissimilarities (Zhou et al., 2015). In the softer cultivar (Toyonoka) PG activity and *PG* transcripts dramatically increased during the last stages of fruit ripening and correlates with fruit softening, whereas in the harder cultivar (Sweet Charlie) a lower level of *PG* transcripts was detected during ripening meanwhile PG activity decreased. The direct relation between PG and fruit firmness in *F.* × *ananassa* was only confirmed after silencing *FaPG1*, showing that transformed fruit had a diminution in pectin solubilization (Pose et al., 2013).

Another enzyme that acts on pectin through a β-elimination reaction is PL. In *F.* × *ananassa* the expression of *PL* increases with the first signs of ripening, and high expression levels are detected in fully ripe fruit, indicating a clear relationship with ripening (Medina-Escobar et al., 1997; Benítez-Burraco et al., 2003). Three *FaPL* genes (*PLa*, *PLb*, and *PLc*) have been reported in strawberry (Benítez-Burraco et al., 2003), although only *FaPLc* showed a clear increment in transcript accumulation during ripening (Severo et al., 2011). The reduction in the expression of *FaPL* in fruit by antisense strategy provided fruit with significant higher firmness, confirming that PL is involved in softening (Jiménez-Bermúdez et al., 2002). Nevertheless, the transgenic lines generated by silencing *FaPG1* showed the preponderant role of PG in fruit firmness, contrary to what was claimed for PL (Pose et al., 2013). In *F. chiloensis* fruit *FcPL* is expressed coincidently with firmness reduction, nevertheless the low level of transcripts and activity indicates a less important role compared to *FcPG* (Figueroa et al., 2008).

PME catalyzes reactions on pectins by double-displacement mechanisms promoting: (1) de-esterification through transferring the C6 carboxyl groups in the pectin-PME complexes to water molecules altering the degree and pattern of methyl esterification, and (2) transacylation through transferring the C6 carboxyl groups to hydroxyl groups of another pectin molecule (Kohli et al., 2015). De-esterification of pectins seems to be necessary for providing access to other pectinases such as PG. PME activity has been detected in growing *F. chiloensis* fruit (early stages of development), and the activity reached a maximum level at the transition stage (Figueroa et al., 2010). It is therefore, considered as an early player during cell wall disassembly. In commercial strawberry it has been suggested a close relationship between PME and softening, because PME activity is reduced immediately after UV-C irradiation with the consequence of a firmer fruit (Pombo et al., 2009). A decrease in the firmness of *F.* × *ananassa* fruit at the late ripening stages is concomitant to the high accumulation of transcripts reported for *PME* and *PG* (Severo et al., 2011).

The metabolism of pectins required an organized performance as a synchronized participation of pectinases is necessary. PME is an early player, followed by PG and/or PL. The major participation of PG in the case of *F. chiloensis* than PL could explain the complete depolymerization of pectins observed in this fruit.

Other pectinases have received much less attention. This is the case of Rhamnogalacturonan I lyase (RGL) (EC 4.2.2.-) that catalyzes the cleavage of α-(1,4) bonds between rhamnose and galacturonic acid in the rhamnogalacturonan I backbone chain of pectins by a β-elimination mechanism (Mutter et al., 1996). Only recently, Molina-Hidalgo et al. (2013) described the participation of RG lyase I in the degradation of RG-I backbone in *F.* × *ananassa*. *FaRGL I* is expressed in fruit receptacles with a maximal accumulation of transcripts in overripe and senescence stages. Gene transient silencing of *FaRGL I* prevents the dissolution of middle lamella (Molina-Hidalgo et al., 2013).

On the other hand, pectins are often ramified by different oligosaccharides, and as described for the principal backbone, a series of enzymes should participate in their metabolism. β-galactosidases and α-arabinofuranosidases are some of the enzymes taking part in the removal of pectin side chains and may act on side chains of pectic (or hemicellulosic) polysaccharides. In commercial strawberry, three β-*Gal* genes were expressed in the fruit, but only *Fa*β*Gal1* showed a high accumulation of transcripts at the ripe stage (Trainotti et al., 2001). The highest accumulation of transcripts takes place at the very late ripening stage (Severo et al., 2011). Total β-*Gal* activity was also detected during development and ripening of commercial strawberry. Not much is known about *AFase* in ripening fruit except for that three cDNAs have been identified in different strawberry cultivars, and that AFase activity was considerably higher in soft cultivars (Rosli et al., 2009). AFase can remove arabinose from pectin side chains and hemicelluloses. It is believed that the removal of side chains from pectins or hemicelluloses can exposed the remaining polymer to the action of other enzymes, and most importantly, it can alter firmness by reducing the integrity of the oligosaccharide matrix.

## Metabolism of Other Cell Wall Polysaccharides

Another group of enzymes participates in hemicellulose metabolism helping cell wall disassembly, but not as first players in softening, such as EGase and XTH (Figure 1). Increasing levels of *EGase* transcripts have been detected during ripening of commercial strawberry (Harpster et al., 1998). The application of heat treatments to strawberries (cv. Selva) causes lower levels of *EGase* expression and a reduction in EGase activity with the consequent arrest in fruit softening (Martínez and Civello, 2008). Nonetheless, the reduction of *EGase* expression level observed in antisense strawberry plants did not influence fruit firmness (Woolley et al., 2001; Palomer et al., 2006; Mercado et al., 2010).

Xyloglucan endotransglycosylase/hydrolase enzyme displays high specificity for xyloglucans (Rose et al., 2002). XTHs belong to family 16 of glycosyl hydrolases and may display either endotransglycosylase (XET; EC 2.4.1.207), endohydrolase (XEH; EC 3.2.1.151), or both activities (Minic and Jouanin, 2006). Two *XTH* genes were identified in *F. chiloensis*, one associated to fruit ripening (*FcXTH1*) and the other to vegetative tissues (*FcXTH2*) (Opazo et al., 2010). A biochemical characterization of FcXTH1 enzyme showed that it displays strict endotransglycosylase activity (Mendez-Yañez et al., 2017). By means of bioinformatics tools, it has been possible to demonstrate a better interaction of FcXTH1 with xyloglucans than with cellulose (Mendez-Yañez et al., 2017), suggesting its active participation in the reorganization of hemicellulose during ripening of *F. chiloensis* fruit. Two *XTH*s were described in *F.* × *ananassa* that were associated to hemicellulose degradation (Nardi et al., 2014), and interestingly, firmer cultivars showed higher accumulation of transcripts of both genes.

Another player is β-xylosidase (β-Xyl) (EC 3.2.1.37), an enzyme that hydrolizes xylo-oligosaccharides releasing xylose units. *Fa*β*Xyl* from *F.* × *ananassa* has been isolated and characterized during fruit ripening (Bustamante et al., 2006b). The level of *Fa*β*Xyl* transcripts rise at the white fruit stage in a softer cultivar (Toyonaka) while in a harder cultivar (Camarosa) the increase in transcripts took place at a later ripening stage (75% red). The activity of the enzyme remains almost constant in Camarosa, but in Toyonaka a constant increment during ripening was determined with maximum activity when the fruit reached full ripeness (Bustamante et al., 2006b).

Expansins on the other hand are proteins with no catalytic activity that probably disrupt the hydrogen bonds between cellulose microfibrils and cell wall matrix polysaccharides, and thereby allow accessibility to cell wall enzymes (McQueen-Mason and Cosgrove, 1994; Rose and Bennett, 1999). In commercial strawberry fruit, seven expansin genes have been identified and the expression profile of each of them characterized in developing fruit (Civello et al., 1999; Harrison et al., 2001; Dotto et al., 2006). In *F. chiloensis* fruit five expansins have been analyzed and the expression level of *FcEXP1*, *FcEXP2* and *FcEXP5* correlates with fruit firmness reduction (Figueroa et al., 2009). The participation of these three gene isoforms suggests functional redundancy, however, a recent bioinformatics approach analyzed the interaction of each of these expansins with different cell wall polysaccharides (Valenzuela-Riffo et al., 2018). The results suggest that expansin proteins can bind not only cellulose but also other cell wall polymers such as xyloglucans of different conformation. The authors conclude that the three FcEXP proteins could be acting on different cell wall domains.

In summary, many enzymes and proteins with different substrate specificity and mechanisms of action participate in the metabolism of cell wall polysaccharides and promote the disassembly of the complex cell wall structure required for fruit softening. The identification and characterization of isoforms for these enzymes increase the complexity of the process nevertheless the use of new tools can give clues on the time of fruit development and ripening of their participation.

## Regulation of Ripening

It is of most relevance the understanding of how the different molecular events related to ripening are coordinated in non-climacteric fruit, as so far it is still a mystery. Moreover, it seems clear now that a wide signaling network should be integrated at hormonal level, metabolic adaptations and transcriptional switches in order to coordinate this complex process. Recently, some regulatory networks based on transcription factors (TF) have been shown to play a central role in fruit development and ripening, as these TFs are able to coordinate the simultaneous expression of a complex network of genes. Understanding how these regulators can modulate development and ripening of fleshy fruits is of utmost importance.

## Hormones Involved in Fruit Ripening

Hormones play essential roles in fruit development and ripening. In climacteric fruit ethylene is the regulator that induces and coordinates almost the entire ripening process; however, in non-climacteric fruit the role of several hormones is an active research area.

In this regard, *F.* × *ananassa* fruit has become a model species of non-climacteric fruit. A complete hormonal profile of *F.* × *ananassa* fruit was reported by Symons et al. (2012) indicating that the levels of auxins and gibberellins (GA) rise early during fruit development, and drop to low levels before color accumulation takes place in the receptacle. The report also confirms previous information that indicated that free and conjugated indole-3-acetic acid (IAA) reach a maximum level at the green fruit stage and subsequently decline (Archbold and Dennis, 1984). On the other hand, abscisic acid (ABA) levels are low at anthesis and gradually rise throughout development and ripening, reaching the highest level at the ripe fruit stage (Symons et al., 2012). Importantly, the first signs of ABA increments coincide with the drop in auxins. In addition, low levels of brassinosteroids are determined during ripening development.

The hormonal profile of auxins indicates a gradual declination in the supply from achenes at the latter stages of growth, which has been implicated long time ago as the basis of fruit ripening (Given et al., 1988). More recently, RNA-seq strategy was used to describe the expression profile of auxins biosynthesis and signaling during development and ripening of *F.* × *ananassa* (Estrada-Johnson et al., 2017). The content of auxins drops by 50% in the receptacle, but remains constant during ripening in a dry weight basis, supporting the idea that auxins could be involved in the ripening of strawberry fruit at later stages (Estrada-Johnson et al., 2017).

It has been shown that auxins delay ripening by modifying the expression of many ripening-associated genes (Given et al., 1988). Most of strawberry ripening-related genes are negatively regulated by auxins, although few auxins up-regulated genes have also been described as summarized in Table 1. Studies performed in several *F.* × *ananassa* cultivars highlighted important hormonal effects on the expression of cell wall genes. No effect on the expression level of *FaPG* and *FaEXP2* was observed after the treatment of strawberries with auxins (Medina-Escobar et al., 1997). The expression levels of *FaRGL1* and *Fa*β*Gal1* were negatively regulated by the application of auxins (Trainotti et al., 2001; Molina-Hidalgo et al., 2013). Genes encoding *FaPL* and *FaEGase* were activated at the onset of strawberry ripening and their expression were reduced by exogenous auxins treatments (Medina-Escobar et al., 1997; Harpster et al., 1998; Civello et al., 1999; Aharoni and O’Connell, 2002). On the contrary, the expression of *FaXTH1* and *FaXTH2* were induced by auxins treatment (Nardi et al., 2014). Indications of gene expression regulation for some of these genes were confirmed by identifying auxin responsive elements in their promoters, such as the case of *FaXTH1* (Nardi et al., 2014). Functional analysis of the promoter fragments of *FaEG1* and *FaEG3* confirm that auxin treatment reduces the expression of GUS as reporter gene (Spolaore et al., 2003). Several recent evidences support the role of ABA as a promoter of strawberry ripening (Jia et al., 2011). The decrease in ABA levels due to the inhibition of *NCED1* (9-*cis*-epoxycarotenoid dioxygenase), a key enzyme in ABA biosynthesis, by fluoridone or by RNAi technology, promoted the arrest of *F.* × *ananassa* development (uncoloured fruit phenotype). This phenotype was rescued by ABA treatment. On the other hand, ABA perception was also evaluated in RNAi fruit of *CHLH/ABAR* (a putative ABA receptor) obtaining the same uncoloured phenotype, although it was not reversed by exogenous ABA (Jia et al., 2011). Similarly, the down-regulation of another ABA receptor (*PYR1*) by RNAi delayed the ripening of strawberry fruits, which was not rescued by exogenous ABA (Chai et al., 2011). Collectively these evidences indicate that ABA biosynthesis and its perception are required for ripening of strawberry fruit.

**Table 1:** Reported effect of different hormonal treatments on the expression of cell wall degrading genes in strawberry fruit.

Hormone	PG	PL	β-Gal	PME	XTH	EGase	EXP	RGL	Xyl
AUX	No effect^1,2^	Reduced expression^3^	Reduced expression^4^	Increased expression^5^	Increased expression of *FcXTH^6^* Increased expression of *FaXTH1* and F*aXTH2*^7^	Reduced expression^3^ Reduced expression of *FaEG1* and *FaEG3*^8^	No effect on *FaEXP*^1^ and *FaEXP2*^9^ Reduced expression of *FcEXP1*, *FcEXP2*, and minor repression of *FcEXP5*^10^	Reduced expression of *FaRGL1*^11^	Reduced expression of *FaXyl1*^12^
ABA					Increased expression of *FcXTH1*^16^ and *FaXTH1*^17^ Reduced expression of *FaXTH2*^7^		Increased expression of *FaEXP2*^9^	Increased expression of *FaRGL1*^11^	Increased expression of *FaXyl1*^12^
GA					Increased expression of *FcXTH1*^6^, *FaXTH1* and *FaXTH2*^7^		No effect on *FaEXP2*^9^		Reduced expression of *FaXyl1*^12^
Eth	Increased expression of *FaPG1*^13,14^ Reduced expression of *FaPG2*^15^	Reduced expression of *FaPLa*^15^	Reduced or no effect on *FaGal*^4^ Increased expression of *FaGal1* and *FaGal2*^14^	Reduced expression of *FaPE1*^14,16^ Increased expression of *FaPE1*^15^	Reduced expression of *FaXTH1* and *FaXTH2*^14^		No effect on *FaEXP2*^9,17^		Reduced expression of *FaXyl1*^12,14^
MeJa					Increased expression of *FcXTH1*^18^	Increased expression of *FcEG1*^18^			

^1^*Medina-Escobar et al. (1997), ^*2*^Harpster et al. (1998), ^*3*^Aharoni and O’Connell (2002) Aharoni et al., 2002, Figueroa et al., 2012., ^*4*^Trainotti et al. (2001), ^*5*^Figueroa et al. (2010), ^*6*^Opazo et al. (2013), ^*7*^Nardi et al. (2014), ^*8*^Spolaore et al. (2003), ^*9*^Nardi et al. (2016), ^*10*^Figueroa et al. (2009), ^*11*^Molina-Hidalgo et al. (2013), ^*12*^Bustamante et al. (2006b), ^*13*^Villarreal et al. (2010), ^*14*^Villarreal et al. (2016), ^*15*^Merchante et al. (2013), ^*16*^Castillejo et al. (2004), ^*17*^Civello et al. (1999), ^*18*^Concha et al. (2013). Hormones are the following: Auxins (AUX), abscisic acid (ABA), gibberellic acid (GA), ethylene (Eth), methyl jasmonate (MeJa)*.

On the other hand, several evidences support that ABA is inducing transcriptional changes in cell wall degrading enzymes. The expression of *FaRGL1* was demonstrated to be positively regulated by ABA (Molina-Hidalgo et al., 2013). Similarly, the expression level of *FaXyl1* increased when ABA was applied, and in contrast *FaXyl1* expression decreased after treatment with ethylene, auxins or gibberellic acid (Bustamante et al., 2006a). The expression of *FaXTH1* is up-regulated in *F.* × *ananassa* fruit in response to ABA and GA treatments, and after achenes removal (Nardi et al., 2014). Recently, Nardi et al. (2016) demonstrates that the promoter sequence of *FaEXP2* drives the expression of a reporter gene toward the receptacle and along ripening. Moreover, *FaEXP2* promoter contains ABA responsive elements, as well as, GA responsive elements.

It has been proposed that ethylene could play a role at early stages of fruit ripening, considering that the level of transcripts for ethylene receptors is high throughout ripening, although the level of ethylene is low (Trainotti et al., 2005). Initially it was reported the increment in the accumulation of *FaPG1* transcripts in response to ethylene (Villarreal et al., 2010), and more recently, several other genes involved in cell wall modification were up regulated by ethylene application such as *FaPG1*, *FaGal1* and *FaGal2* (Villarreal et al., 2016). On the contrary, genes such as *FaPME1*, *FaXyl1*, *FaXTH1* and *FaARA1* were down regulated in response to ethylene, meanwhile *FaPLa* did not show any change at all (Villarreal et al., 2016). Others reported the down regulation of *FaPG2* and *FaPLa*, the up-regulation of *FaPE1*, and no changes in *FaPG1* in ethylene treated strawberry (Merchante et al., 2013). Additionally, all three *Fa*β*Gal* genes were down regulated in response to ethylene treatment (Trainotti et al., 2001). Interestingly, three putative ethylene-responsive elements were found in the promoter region of *FaPE1* (Castillejo et al., 2004).

Little is presently known about the hormonal control of *F. chiloensis* fruit. The application of auxins induces the expression of *FcPME* and *FcXTH1* (Opazo et al., 2013). Meanwhile auxins strongly repress the expression of *FcEXP1* and *FcEXP2*, and have a minor repression effect on *FcEXP5* (Figueroa et al., 2009). Furthermore, the removal of achenes (the endogenous source of auxins) promotes the increase in *FcEXP2* expression in *F. chiloensis* (Figueroa et al., 2009), in agreement with a recent report in *F.* × *ananassa* (Nardi et al., 2016). This suggests a negative effect of auxin on *EXP2* expression. On the other hand, the treatment of *F. chiloensis* fruit with ABA and GA has an activator effect on the transcription of *FcXTH1* (Opazo et al., 2013). The analysis of FcXTH1 promoter sequence reveals different regulatory elements responding to hormones, which could explain the accumulation of transcript pattern (Opazo et al., 2013). In the case of jasmonic acid little information is available, but recently it was reported that the accumulation of *FcXTH1* and *FcEG1* transcripts increased after the application of methyl jasmonate (Concha et al., 2013). Finally, the blockage of ethylene perception by 1-methylcyclopropene (1-MCP) repressed the expression of *FcXTH1* in *F. chiloensis* fruit, and in concordance with that, ethylene responsive elements are present in FcXTH1 promoter (Opazo et al., 2013).

Although the knowledge concerning the hormonal regulation of strawberry fruit is growing, it is still limited. So far, it is clear that the levels of auxins and ABA in the receptacle, or even more precisely the relation ABA/AUX, may have an important role in the coordination of fruit development and ripening in strawberry fruit (Figure 1) as it has been suggested by Medina-Puche et al. (2016). In the case of softening of strawberry fruit, key genes are regulated by ABA, which tells us that this hormone could be the main modulator of gene expression during ripening of this non climacteric fruit.

## Transcription Factors and Fruit Ripening

If little is known regarding the interactions between different hormones on fruit ripening and softening in strawberry fruit, the role of transcription factors (TF) in this process is still in its infancy. It is clear that TFs mediate changes in the expression of genes responsible for the complete program of cellular events required for the development and ripening, but one of the problems to be solved during the study is that many TFs belong to a family of proteins, and therefore it is difficult to clarify the role of a single TF. In addition to that, several TFs from different families interact forming active transcriptional complexes that complicate even more the study. Even though, notable results have been obtained within the synthesis of flavonoids in strawberry fruit, where three TFs (MYB, WD40 and bHLH) interact in the transcriptional control of anthocyanins biosynthesis (Schaart et al., 2012; Salvatierra et al., 2013). Interestingly, even if the description of FaGAMYB was mainly focus in secondary compounds, the results indicated that *FaPE*, *FaPG* and *FaPL* were down-regulated in *FaGAMYB* silenced strawberry (Vallarino et al., 2015), suggesting also a role of *FaGAMYB* in the remodeling of cell wall.

FaDOF2 is another TF, which is expressed preferentially in the receptacle of strawberry during ripening and senescence, but low level of transcripts were detected at early stages of fruit development (Molina-Hidalgo et al., 2017). Moreover, it was shown that ABA induces the expression of *FaDOF2* meanwhile auxins repress it. Strawberry fruit were transformed with interfering *FaDOF2* mRNA, and changes in gene expression were studied using the microarray FraGenomics 35K. Albeit the limitations of using the array, several genes modified their transcriptional levels in *FaDOF2* silenced strawberry. Transcription factors like MYB, NAC, MADS and GRAS showed altered gene expression. Most interestingly, several genes associated to cell wall modification also showed up or down regulation, like: *PE*, *PME*, *XTH*, *PG1*, *PG2*, *EXP*, β-*Glu* and *PL* (Molina-Hidalgo et al., 2017). Clearly, *FaDOF* is acting at the late stage of ripening and senescence and modulates the expression of several genes, but further analysis should give clues about TF partners or if it is directly involved in softening.

MADS-box is a large family of TFs associated to floral development, as well as, to fruit development and ripening (Shore and Sharrocks, 1995; Ito et al., 2008; Díaz-Riquelme et al., 2009; Gramzow and Theissen, 2010; Seymour et al., 2011; Ireland et al., 2013). In *F.* × *ananassa* the suppression of *FaMADS9* expression (homologous to SEPALLATA1/2) produces defects in petal formation, as well as, in achenes and the receptacle (Seymour et al., 2011). Albeit, MADS-box TFs are able to change the anthocyanin content and color of the fruit (Seymour et al., 2011), the series of genes regulated by MADS-box TFs are still unrevealed. Interestingly, *FaMADS1a* is expressed with delayed ripening phenotype, and its expression is induced by auxin and suppressed by ABA, concomitantly speeding up the ripening process (Lu et al., 2018). In *F. chiloensis* two MADS-box genes (*FcMADS1* and *FcMADS2*) have been identified, and both genes are expressed during fruit ripening, but also highly expressed in flower tissues (Pimentel et al., 2010).

NAC is another TF expressed during strawberry ripening. A large number of NACs were recently identified in *F.* × *ananassa*, six of them expressed during fruit development and ripening (Moyano et al., 2018). ABA positively regulates the expression of *FaNAC*, meanwhile auxins inhibit the expression of this TF (Moyano et al., 2018). In the case of *F. chiloensis* fruit, a NAC transcription factor has been identified and analyzed. *FcNAC1* is expressed during fruit ripening and its expression is suppressed after the addition of auxins (Carrasco-Orellana et al., 2018). Moreover a fast accumulation of *FcNAC1* transcripts are observed after ABA treatment, although this increment was promptly reduced (Carrasco-Orellana et al., 2018). More interestingly, FcNAC1 promoter region showed response elements for several plant hormones, including ABA, MeJa, auxin and gibberellic acid (Carrasco-Orellana et al., 2018). Additionally, the isolated promoter region of *FcPL* was transactivated by FcNAC1 in a luciferase assay, and on the contrary, the promoter region of *FcEXP2* was not transactivated by FcNAC1. This suggests that *FcNAC1* could play an important role in the transcriptional regulation of cell wall degrading genes.

## Conclusion

Thus, fruit softening is an extremely complex process that requires the contribution of multiple enzymes and proteins that sequentially and in a coordinated way modify the structure of the cell wall. As many proteins are required and each of them needs to get into action at a particular time during fruit development, the precise organization of the entire process is unquestionably. Hormones like ABA and auxins, and more precisely the relation ABA/AUX, have a crucial role in the coordination of softening events in *F.* × *ananassa* and *F. chiloensis* fruit. The coordination of hormones regulates the expression of many cell wall degrading enzymes and the massive transcriptional change that takes place involves several TFs. We predict that the entire process is even more complex than expected, many more players are about to be identified and therefore more efforts are needed to fill the gaps. Even though, we don’t know with certainty how the ripening of strawberry fruit is regulated, the entire process is still under active research especially for the great impact of texture on fruit quality and their high impact on fruit shelf life.

## Author Contributions

RH and MM-L conceived and designed the study. EM-A contributed in collecting information and preparing figures. RH, MM-L, and EM-A wrote and drafted the manuscript. All authors participated sufficiently in the work to take public responsibility for appropriate portions of the content, and read, edited, and approved the final manuscript.

## Conflict of Interest Statement

The authors declare that the research was conducted in the absence of any commercial or financial relationships that could be construed as a potential conflict of interest.
